# Quantitative ethnomedicinal study of plants used in the skardu valley at high altitude of Karakoram-Himalayan range, Pakistan

**DOI:** 10.1186/1746-4269-10-43

**Published:** 2014-05-09

**Authors:** Abida Bano, Mushtaq Ahmad, Taibi Ben Hadda, Abdul Saboor, Shazia Sultana, Muhammad Zafar, Muhammad Pukhtoon Zada Khan, Muhammad Arshad, Muhammad Aqeel Ashraf

**Affiliations:** 1 Department of Plant Sciences, Quaid-i-Azam University, Islamabad 45320, Pakistan; 2 School of Chemical Engineering, Universiti Sains Malaysia, Nibong Tebal 14300, Malaysia; 3 Laboratoire de Chimie des Matériaux, Faculté des Sciences, Université Mohammed Premier, Oujda 60000, Morocco; 4 Department of Agricultural Economics, PMAS-Arid Agriculture University, Rawalpindi, Pakistan; 5 Department of Botany, PMAS-Arid Agriculture University, Rawalpindi, Pakistan; 6 Department of Geology, University of Malaya, 50603 Kuala Lumpur, Malaysia

**Keywords:** Quantitative ethnobotany, Medicinal plants, Skardu valley, Northern Pakistan

## Abstract

**Background:**

The tribal inhabitants of the Skardu valley (Pakistan) live in an area of great endemic botanic diversity. This paper presents the first quantitative ethnomedicinal spectrum of the valley and information on the uses of medicinal plant. This paper aims to analyze and catalogue such knowledge based on Relative Frequency Citation (RFC) and Use Value (UV) of medicinal plants in addition to the configuration of the Pearson correlation coefficient.

**Methods:**

The field study was carried out over a period of approximately 2 years (2011–2013) using semi-structured interviews with 71 informants (most of the informants belonged to an age between 50 and 70 years) in six remote locations in the valley. Ethnomedicinal data was analyzed using frequency citation (FC), relative frequency of citation (RFC) and use value (UV) along with a Pearson correlation coefficient (PCC). Demographic characteristics of participants, ethnobotanical inventory of plants and data on medicinal application and administration were recorded.

**Results:**

A total of 50 medicinal plants belonging to 25 families were reported to be used against 33 different ailments in the valley. The maximum reported medicinal plant families were Asteraceae (7 report species), Lamiaceae (6) , Polygonaceae (4) and Rosaceae (4), the most dominant life form of the species includes herbs (38) followed by shrubs and subshrubs (12), the most frequent used part was leaves (41%) followed by root (26%), flower (14%), fruit (9%), seeds (8%), bulb (1%) and bark (1%), the most common preparation and administration methods were infusion (32%), decoction (26%), paste (18%), herbal juice (17%) and powder drug (7%). The Pearson correlation coefficient between RFC and UV was 0.732 showing highly positive significant association.

**Conclusions:**

In this study, we have documented considerable indigenous knowledge about the native medicinal plants in Skardu valley for treating common ailments which are ready to be further investigated phytochemically and pharmacologically which leads to natural drug discovery development. The study has various socioeconomic dimensions which are associated with the local communities.

## Background

Pakistan is rich in diversity of medicinal and aromatic flora due to its unique phytogeography with diverse climatic conditions. About 400–600 medicinal plant species out of 5700 are estimated to exist in Pakistan. In early 1950 almost 84% of the Pakistani population mainly dependent on traditional medicines as a primary health care source [1] but nowadays this dependency is limited only to the remote areas due to rapid change in lifestyle and modernization [2]. However, the Northern Pakistan is still considered to be one of the richest regions in the country in terms of its biodiversity and culture of utilization of unique medicinal plant resources [3]. The three mountain ranges, the Himalaya, the Karakorum and the Hindu Kush (HKH) collectively contain about 25,000 species (about 10% of world plant species), out of which around 10,000 are economically or medicinally valuable [4]. These mountainous regions provide a naturally conducive environment for the growth of medicinal flora. People living in these regions of Pakistan are using plants in many ways like medicines, timber wood, firewood, food and fodder [5]. In Himalayan ranges about 70% of the medicinal flora and animals are used as wild species, 70-80% of the inhabitants are dependent on traditional medicines using plants to cure their common ailments [6]. These regions are rich with endemic species and around 1,000 species of vascular plants are known to occur in the northern mountain regions of Pakistan [7-9]. Alam [10] reported eight endemic species from Gilgit Baltistan, out of these *Astragalus clarkeanus, Asperula oppositifolia* subsp. *baltistanica, Berberis pseudoumbellata* subsp. *gilgitica, Haplophyllum gilesii* and *Tanacetum baltistanicum* are found critically endangered (CR) while *Aconitum violaceum var. weileri* and *Rhodiola saxifragoides* are vulnerable (VU)*.*

Generally in Deosai plateau, about 342 species of plants belonging to 36 families and 142 genera have been recorded in the flora of Pakistan so far, while to the best of our knowledge, the number of species used as medicinal are not systematically recorded in literature. This high level of biodiversity on the plateau is due to several reasons, including topography, location of the plateau (Junction of major mountain ranges) and local adaptation of its plant and animal species [11]. Sultana et al. [12] reported 43 species of Poaceae, 32 species of Cyperaceae and 4 species of Juncaceae from Deosai plateau. A total of 114 plant species belonging to 28 families were found around the Sheosar Lake of Deosai plateau [13]. These studies on Deosai [11-13] were based on biodiversity, altitudinal distribution of species and phytosociological expeditions. The present study focuses only on medicinal plant species frequently used by the local populace of Skardu valley. The study area has a rich potential for utilization and consumption of medicinal and aromatic plants. These areas are also the potential sites for exporting tradable medicinal plants on a sustainable basis [14].

Numbers of ethnobotanical studies on the use of medicinal plants in traditional health care were carried out in the neighboring parts of these ranges by previous ethnobotanists [14-20]. Whereas, Skardu valley was neglected all the time, due to its rough geography, high altitude and strict cultural and religious bans for outsiders to document such source of knowledge. There is a typical social and economic fabric of this region which is highly associated to the flora and fauna available in the valley. People give much importance to the plants and trees for their domestic and medicinal uses. An in depth study has not been pursued in the past. To fill up this gap, the folk medicinal uses of the plants of the valley were documented in the form of an ethnomedicinal inventory. Specifically the main aims of this investigation are: (i) to compile the ethnoflora with traditional medicinal applications of the Skardu valley for the scientific community, (ii) to assess quantitatively the ethnomedicinal data using RFC and UV indices in order to look for most cited and used species which have not been previously reported or with limited past reports on medicinal uses that may provide baseline data for future evaluation regarding their pharmacological and clinical screening.

## Materials and methods

### An overview of the geography and climate of the study area

**Figure 1:**
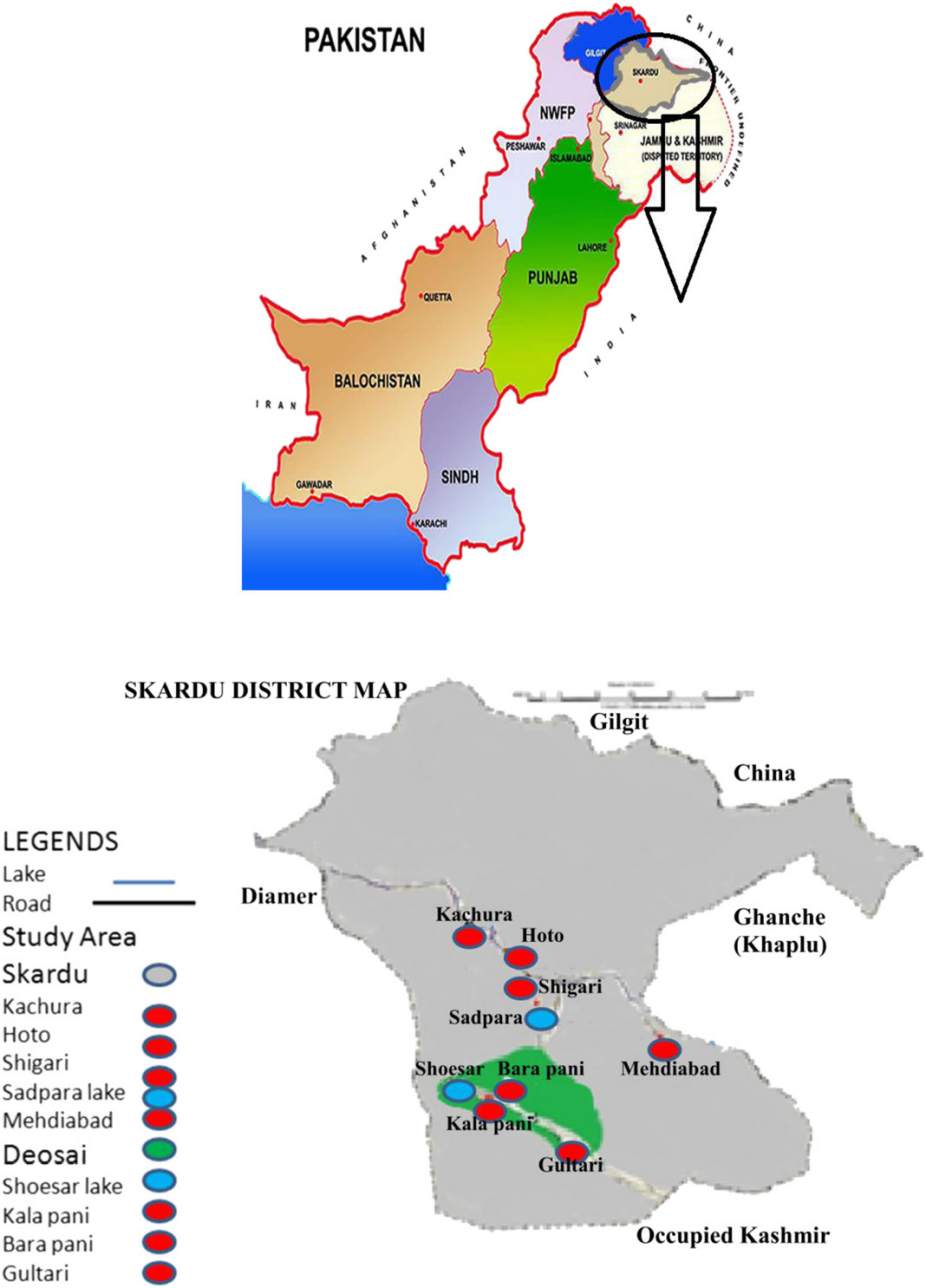
Map of District Skardu showing the study sites.

The Karakoram Range covers the borders of India, China and Pakistan (in the regions of Gilgit-Baltistan). The Himalaya range in Pakistan occupies the regions of Deosai, Chilas, Kaghan, Kohistan and Kashmir. Many of the peaks of this range are above 8000 m as Godwin Austin/K-2 (8611 m), Nanga Parbat (8126 m), Gasherbrum I (8080 m), Broad Peak (8051 m) and Gasherbrum II (8035 m). Outside the Arctic, world’s largest glacier, Siachin (78 km long), is located here with temperature close to −50°C. Skardu valley is located at 35° 18' north latitude and 75° 37' east longitude in Baltistan region, Northern Pakistan. Temperatures in the base of valley range from nearly 38°C in summer to less than −25°C in winter. Skardu borders District Ghanche to the east, Indian-occupied Kashmir to the south, the Chinese province of Sinkiang to the north and the Deosai Plateau to the southwest. The Deosai Plateau (30°00 N 75°30 E) is located in the north of the main Himalayan range. Its height is about 4,115 m (14,500 feet) above the timberline and is considered to be one of the highest plateaus of the world. For over half the year (between September – May), Deosai is snow covered and inaccessible to visit (snow is 7–8 yard deep). Deosai is accessible from Skardu in the north and the Astore valley in the west. It is about 30 km from Skardu and covers an area of almost 5,000 km^2^. The various sites of Deosai Plateau are Shatun Nala, Bara Pani, Kala Pani, Murtaza Camp, Burzil Top, Shausar Lake, Gultari, Chota Deosai, Barila, Chilam Chauki, Farenshot and Dumbo Bahao. This ethnomedicinal exploration was confined to nine botanically rich sites of the valley includes Kachura, Hoto, Shigari, Sadpara Lake, Mehdi Abad and four selected sites of Deosai plateau (Shausar lake, Bara pani, Kala pani, Gultari) (Figure 1). As our focus is on the most frequently used medicinal plants, the local herbalist and laymen having rich traditional knowledge preferred these sites for medicinal plant collection. Elevations of the selected study sites range from approximately 2,270 m to 4,115 m on the Deosai Plains. The flora of the valley is rich and diverse due to its altitude, unique climate and other topographic conditions [21].

### Ethnomedicinal data collection and ethnographic composition

Field work consisted of data documentation, plant collection and photography. Prior rural appraisal (PRA) approach was adopted according to Kyoto protocol concerning the intellectual property rights of local inhabitants and plant resources of the area. Formal written consent, including consent for publication was received from all the informants before the interviews began. The method employed during the study was designed with the sole purpose for eliciting the precious wealth of local knowledge on medicinal plant use [22]. A total of 71 informants with a different age range were randomly selected for interviews. A total of 35 men (49%), 30 women (42%) and men traditional healers 11 (15%) were interviewed. The informants were divided into two age groups (1) 41–60 and (2) 61–80 years old. Most of the informants belonged to an age between 50 and 70 years. The ethnic composition of the valley is quite diverse, mainly resides with Baltistan Mongle, Mon, Hor, Brokpa and Kashmiri. Primary local language spoken in the area is Balti (90% of the population) while the other locally known languages include Shina, Burshishki and Wakhi.

The selection of informants was mainly based on their rich indigenous knowledge and long term experience of utilization of plants. Field surveys mainly comprised, general meeting, male interviews, female interviews, local herbalists (Hakims) interviews and transect walk. The semi-structured questionnaire based interviews were begun with informants after explaining the aims of the study. The informants were asked various questions about their traditional knowledge, plant use, disease treated, part used and the method of preparation and administration. Interviews were generally conducted in the local language of Balti at village male gathering places, mosques and sometimes in houses. In order to collect detailed information relating to herbal medicine inhabitants of the community were requested to share the knowledge of medicinal plant utilization in local language. All interviews were conducted with the assistance of native translators in their local language. All documented data were then translated into English.

Data on local names of the plants, part used, method of preparation, administration route, and application to treat diseases was recorded. In some cases field photos of the medicinal plants were used to confirm the local names by the informants. The plant specimens were collected during field survey with the help of informants. The medicinal plants were identified using regional Flora [8] and plant taxonomists at the Herbarium of Pakistan (ISL) Quaid-i-Azam University.

### Socio-economic conditions of Skardu valley

The flora of the Skardu valley is very diverse with hundreds of medicinal plants with useful pharmaceutical values and a number of economically important species of wild plants. Biodiversity is a significant natural resource for the socio-economic welfare of the people of the valley. Wild plant species and traditional medicine were one of the economical sources for the local communities. Animal and plant communities supported the development of early populaces of this region for centuries, providing the base for the evolution from hunting to forestry, agriculture, animal husbandry and now tourism and trade. The narrow valley and high mountains kept Skardu physically isolated which forced indigenous people to depend on local biodiversity for food and other essential needs. Continuing use and maintenance of biological diversity is important to the people of Skardu. Economic development of Skardu valley depends on management of high diversity of crops, maintaining high pastures, raising fodder species in varied mountain environment and development of livestock biodiversity and medicinal plants. These will be the feasible options for ensuring food security and generating cash income of the people of the valley.

### Quantitative ethnomedicinal data analysis

#### Relative Frequency Citation (RFC)

The collected ethnomedicinal information was quantitatively analyzed using an index of relative frequency citation (RFC) as;

$$\mathrm{RFC}=\mathrm{FC}/N\;\left(0<\mathrm{RFC}<1\right)$$

This index shows the local importance of each species and it is given by the frequency of citation (FC, the number of informants mentioning the use of the species) divided by the total number of informants participating in the survey (N), without considering the use-categories [23].

#### Use Value (UV)

The Use Value (UV) demonstrates the relative importance of plants known locally. It was calculated using the following formula [24].

UV=∑Ui/N

Where Ui is the number of uses mentioned by each informant for a given species and N is the total number of informants.

#### Pearson correlation coefficient

Pearson product–moment correlation coefficient is a good measure to numerically quantify the nature of the linear relationship between two variables. Pearson’s correlation coefficient is the ratio of the covariance between two variables to their standard deviations and calculated from a sample as

(1) $$r=\frac{\mathrm{COV}\left(X,Y\right)}{\mathrm{SD}\left(X\right)*\mathrm{SD}\left(Y\right)}=\frac{E\left(X-\bar{X}\right)\left(Y-\bar{Y}\right)}{\mathrm{SD}\left(X\right)*\mathrm{SD}\left(Y\right)}$$

Where r is the Pearson correlation coefficient for the given sample, COV is the covariance, X and Y are the variables for which we are interested to explore the relationships and SD is the standard deviation for the same variables and calculated as

(2) $$\mathrm{SD}\left(X\right)=\sqrt{\frac{1}{n-1}\sum_{i=1}^{n}{\left(X_{i}-\bar{X}\right)}^{2}}$$

(3) $$\bar{X}=\frac{\sum_{i=1}^{n}X_{i}}{n}$$

Where X bar is the mean value of X and n is the sample size [25]. Similarly SD(Y) can be estimated. To test the null hypothesis of no linear relationship between the variables (ρ = 0), z-test was used at the 5 percent level of significance. The value of z-stat can be obtained by

(4) $$z=\frac{\sqrt{n-3}}{2}\ln\left[\frac{\left(1+r\right)\left(1-\rho \right)}{\left(1-r\right)\left(1+\rho \right)}\right]$$

Where ρ is the population coefficient. If the P - value of z-stat is less than 5 percent, it means the two variables have a significant linear association with each other [26]. In our case the two variables of interest are the RFC and UV. Since the correlation is the quantitative measure of the degree to which patterns RFV and UV respond while RFC vary across different species as do UV. The correlation squared (r2) measures the cross species variability in RFC that is explained by the variance in UV and is obtained simply by taking the square of the r [27].

### Ethical approval

This ethnomedicinal study was duly approved by the ethical committees of the Herbarium (ISL), Department of Plant Sciences Quaid-i-Azam University, Islamabad and Biodiversity Action Plan (BAP-2010-2020) for Pakistan. Prior permission was sought from the Forest Department, Gilgit-Balistan Region for the collection of plants and ethnomedicinal data in the area. PIC was obtained from the informants after elaborating the aims of the survey Table 1.

**Table 1:** Use reports

**Botanical name and voucher number**	**Use reports**
*Allium humile* Kunth	5
SK 103	
*Carum carvi* L.	3
SK 104	
*Heracleum candicans* Wall. ex DC.	2
SK 105	
*Artemisia sieversiana* Ehrh.	3
SK 108	
*Cichorium intybus* L.	3
SK 109	
*Jurinea dolomiaea* Boiss	4
SK 110	
*Pseudognaphalium luteoalbum* (L.) Hilliard & B. L. Burtt.	1
SK 111	
*Senecio chrysanthemoides* DC.	3
SK 112	
*Seriphidium brevifolium* ( Wall. ex DC. ) Ling & Y.R.Ling	1
SK 107	
*Tanacetum gracile* Hook. f. & Thoms.	2
SK 113	
*Berberis vulgaris* L*.*	3
SK114	
*Lepidium latifolium* L.	3
SK 115	
*Codonopsis clematidae* C.B. Clarke	2
SK 116	
*Capparis spinosa* L.	5
SK 117	
*Chenopodium album* L.	5
SK 118	
*Chenopodium botrys* L.	5
SK 119	
*Rhodiola imbricata* Edgew.	4
SK 120	
*Juniperus excelsa* M. Bieb*.*	4
SK 121	
*Hippophae rhamnoides* L.	4
SK 122	
*Ephedra gerardiana* Wall.	5
SK 123	
*Swertia petiolata* Royle	4
SK 124	
*Gentiana olivieri* Griseb.	1
SK 125	
*Geranium nepalense* Sweet	5
SK 126	
*Dracocephalum nuristanicum* Rech. f. & Edelb.	1
SK 106	
*Mentha royleana* Wall. ex Benth	2
SK 127	
*Perovskia abrotanoides* Kar.	4
SK 128	
*Nepeta leucolaena* Benth. ex Hook.f	2
SK 129	
*Thymus linearis* Benth.	4
SK 130	
*Prunella vulgaris* L.	4
SK 131	
*Rhododendron anthopogon* D. Don.	4
SK 132	
*Epilobium latifolium* L.	4
SK 133	
*Astragalus psilocentros* Fisch	2
SK 134	
*Sophora mollis* Graham	1
SK 135	
*Plantago lanceolata* L.	5
SK 136	
*Bistorta amplexicaulis (*D.Don.) Greene	3
SK 137	
*Oxyria digyna* (L.) Hill.	3
SK 138	
*Polygonum affine* D. Don.	4
SK 139	
*Rheum australe* D. Don	6
SK 140	
*Primula denticulata* Smith	4
SK 141	
*Aconitum violaceum* Jacquem. ex Stapf	5
SK 142	
*Caltha alba* Jacquem*.*	2
SK 143	
*Delphinium brunonianum* Royle	4
SK 144	
*Potentilla argyrophylla* Wall. ex Lehm. var. *atrosanguinea* (G.Lodd.) Hook.f.	1
SK 145	
*Potentilla bifurca* L.	1
SK 146	
*Potentilla salesoviana* Steph.	4
SK 152	
*Rosa brunonii* Lindl.	1
SK 147	
*Bergenia stracheyi* (Hook. f. & Thorns.) Engl.	6
SK 148	
*Bergenia ciliata* (Haw.) Sternb.	5
SK 149	
*Pedicularis cheilanthifolia* Schrenk	2
SK 150	
*Pedicularis pectinatiformis* Bonati	2
SK 151	

## Results and discussion

### Medicinal plant diversity, part used and administration methods

**Figure 2:**
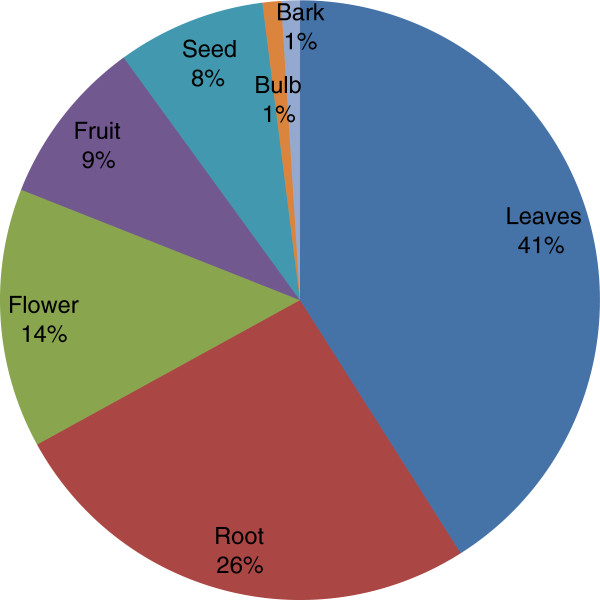
Percentage of plant parts used in herbal preparations.

**Figure 3:**
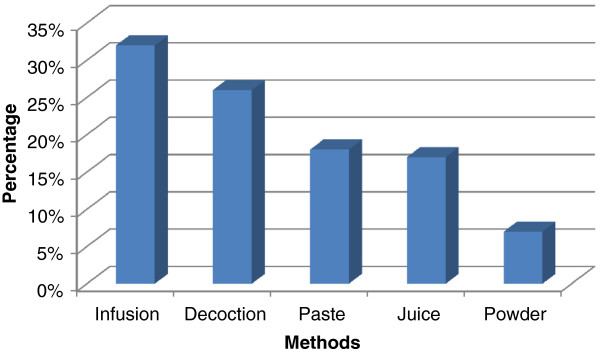
Preparation methods of herbal medicine.

**Figure 4:**
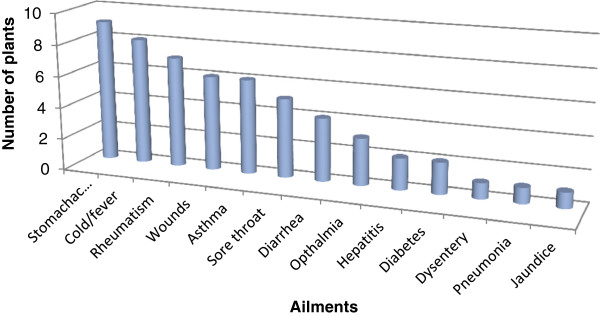
Number of plants used to treat various diseases.

A total of 50 plant species belonging to 44 genera of 25 families were catalogued in Table 2, with traditional uses as herbal medicine against various diseases. The study was carried out in nine selected sites of the valley to document the traditional knowledge of local people on medicinal plants. The most encountered medicinal plant families were Asteraceae (7 reported species), Lamiaceae (6), Polygonaceae (4) and Rosaceae (4) while the rest of the family was represented with variable number of less than 4 species. Our findings regarding the predominance utilization of family Asteraceae and Lamiaceae in the Skardu valley were in agreement with other ethnic-floras [28-31]. The reasons for high degree of ethnomedicinal plants of families Asteraceae and Lamiaceae in our region are due to their wide occurrence with a number of traditional uses known by the local informants. The most dominant life form of the species reported includes herbs (38) followed by shrubs and subshrubs (12). A similar pattern of life form was reported in ethnobotanical survey of our neighboring country India [32]. The parts of the plant primarily used are the leaves (41%), while roots (26%), flowers (14%), fruits (9%), seeds (8%), bulbs and barks (1%) are also frequently used (Figure 2). These reports were in agreement with previous studies conducted in different parts of the world, where the leaves are cited as commonly used parts of the medicinal plants. Medicinal plants used in folk herbal remedies are prepared and administered in various forms in the valley. The most common preparation and administration methods were categorized into infusion (32%), decoction (26%), paste (18%), herbal juice (17%) and powdered drug (7%) (Figure 3). However, these results are different in comparison with previous ethnobotanical studies [33,34]. The percentage of oral administration (77%) of herbal preparation is almost higher than external or topical (23%) application. Similar to our findings regarding the administration route of herbal drugs, the oral administration was also reported in previous work conducted in Bolivia [35]. The most treated illnesses of the Skardu valley using a number of medicinal plants are grouped into 33 pathological disorders. We found the highest number of plant species are used against stomachache (9 species) followed by cold/fever (8 species), rheumatism (7 species), wounds (6 species), Asthma/breathing problem/difficulty in breathing (6 species), sore throat (5 species), diarrhea (4 species), eye diseases (3 species), hepatitis and diabetes (2 species each) (Figure 4). While some other treated diseases includes dysentery, pneumonia, respiratory disorders, jaundice, renal disease, lung disorders, liver disease etc. The local herbalist/Hakims (Person with indigenous knowledge on diseases, its diagnosis and folk treatment) diagnose ailments by their signs and symptoms rather than specific laboratory tests, as this knowledge travel through generations. Figure 4 shows the most treated disease using plants in this study include stomach disorder followed by cold & fever. The findings regarding number of plants used against each disease category were dissimilar to previous work in our neighboring regions [36]. To the best of our knowledge, this variation in comparison to previous studies is additional and novel ethnomedicinal information reported from this valley. This valuable information would create a sense of social and economic ownership of unique floral resources which can help in the preservation of important plant species. By realizing the importance of their natural resources, they can provide a recreational look to the attraction of tourists in the region.

**Table 2:** Medicinal plants used to treat various diseases in Skardu valley

**Family**	**Botanical name and voucher number**	**Local name**	**Life form**	**Parts used/ Formulation**	**Applications**	**Administration route**	*FC	**RFC	***∑Ui	****UV
Alliaceae	*Allium humile* Kunth SK 103	Chung	Herb	Bulb infusion	Asthma/breathing problem	Oral	13	0.18	27	0.38
					Stomach disease					
					Jaundice					
					Cough, cold					
Apiaceae	*Carum carvi* L. SK 104	Zera	Herb	Seed decoction	Gastric problems	Oral	21	0.29	45	0.63
					Sore throat					
					Rheumatism					
Apiaceae	*Heracleum candicans* Wall. ex DC. SK 105	Ghang	Herb	Leaves decoction	Leucoderma	Oral	14	0.19	29	0.40
					Psoriasis					
Asteraceae	*Artemisia sieversiana* Ehrh. SK 108	Khampa	Subshrub	Flower infusion	Pneumonia	Oral	33	0.46	81	1.14
				Leaves decoction	Joints	Oral				
				Root paste	Boils	Topical				
Asteraceae	*Cichorium intybus* L. SK 109	Shantha	Herb	Root infusion	Fever	Oral	22	0.30	54	0.76
				Leaves juice	Release of gall stones	Oral				
					Gastrointestinal problems					
Asteraceae	*Jurinea dolomiaea* Boiss SK 110	Sathing	Herb	Root decoction	Cardiac	Oral	7	0.09	9	0.12
				Leaves juice	Colic	Oral				
				Root poultice	Fever	Topical				
					Eruptions					
Asteraceae	*Pseudognaphalium luteoalbum* (L.) Hilliard & B. L. Burtt. SK 111	Thliri	Herb	Leaves juice	Asthma/breathing problem	Oral	14	0.19	36	0.50
Asteraceae	*Senecio chrysanthemoides* DC. SK 112	Api mindoq	Herb	Leaves decoction	Sore throat	Oral	15	0.21	31	0.43
				Flower poultice	Wounds	Topical				
				Root powder	Rheumatic pain	Topical				
Asteraceae	*Seriphidium brevifolium* ( Wall. ex DC. ) Ling & Y.R.Ling SK 107	Bursay	Subshrub	Flower powder	Expel worms	Oral with water	27	0.38	63	0.88
Asteraceae	*Tanacetum gracile* Hook. f. & Thoms. SK 113	Serfo bursay	Herb	Leaves decoction	Intestinal worms	Oral	9	0.12	19	0.26
				Leaves powder	Obesity					
Berberidaceae	*Berberis vulgaris* L*.* SK114	Shuturum	Shrub	Fruit juice	Sore throat	Oral	38	0.53	72	1.01
					Gastrointestinal pain					
					Remove gall stone					
Brassicaceae	*Lepidium latifolium* L. SK 115	Chunma	Herb	Leaves infusion	Liver disease	Oral	10	0.14	23	0.32
				Root powder	Kidney disease	Oral with water				
					Stomachache					
Campanulaceae	*Codonopsis clematidae* C.B. Clarke SK 116	Bajo mindoq	Herb	Flower infusion	Stress relief	Oral	11	0.15	27	0.38
					Memory retention					
Capparidaceae	*Capparis spinosa* L. SK 117	Shorot	Shrub	Fruit juice	Joint pain	Oral	24	0.33	104	1.46
					Diuretic					
					Dropsy					
				Root decoction	Anemia	Oral				
					Kidney disinfectants					
Chenopodiaceae	*Chenopodium album* L. SK 118	Snew	Herb	Leaves infusion	Rheumatism	Oral	17	0.23	32	0.45
				Leaves poultice	Swollen feet	Topical				
					Sunstroke					
					Sunburn					
					Freckles					
Chenopodiaceae	*Chenopodium botrys* L. SK 119	Khama	Herb	Whole plant infusion	Stomachache	Oral	18	0.25	17	0.23
					Liver complaints					
					Headache					
					Laxative					
					Diuretic					
Crassulaceae	*Rhodiola imbricata* Edgew. SK 120	Chundol	Herb	Root powder	Cough, fever	Oral with milk	11	0.15	38	0.53
					Headache					
					Anemia					
Cupressaceae	*Juniperus excelsa* M. Bieb*.* SK 121	Hlashuk	Shrub	Fruit juice	Remove kidney stone	Oral	37	0.52	66	0.92
					Rheumatism					
					Respiratory disorders backache					
Elaeagnaceae	*Hippophae rhamnoides* L. SK 122	Choq	Shrub	Fruit juice	Cough	Oral	65	0.91	117	1.64
					Arthritic pain					

					Burns					
					Eczema					
Ephedraceae	*Ephedra gerardiana* Wall. SK 123	Chay	Shrub	Stem decoction	Rheumatism	Oral	31	0.43	98	1.38
					Syphilis					
				Fruit juice	Respiratory disorders	Oral				
					Asthma/breathing problem					
					Tonic					
Gentianaceae	*Swertia petiolata* Royle SK 124	Brama	Herb	Root paste	Opthalmia	Topical	15	0.21	33	0.46
				Leaves decoction	Scleritis	Oral				
					Stomach Inflammation					
					Liver tonic					
Gentianaceae	*Gentiana olivieri* Griseb. SK 125	Tikta	Herb	Fresh leaf	Diabetes	Oral	22	0.30	16	0.22
Geraniaceae	*Geranium nepalense* Sweet SK 126	Bamik	Herb	Fruit juice	Renal disease	Oral	44	0.61	43	0.60
				Root poultice	Rheumatic pain	Topical				
					Toothache					
					Wounds					
					Cuts					
Lamiaceae	*Dracocephalum nuristanicum* Rech. f. & Edelb. SK 106	Shamdun	Herb	Seed infusion	Gastrointestinal disorders	Oral	41	0.57	74	1.04
Lamiaceae	*Mentha royleana* Wall. ex Benth SK 127	Foling	Herb	Leaves infusion	Dysentery	Oral	26	0.36	41	0.57
					Abdominal pain					
Lamiaceae	*Perovskia abrotanoides* Kar. SK 128	Faring bursay	Subshrub	Flower infusion	Expel worms	Oral	45	0.63	69	0.97
					Leishmaniasis					
					Rheumatic pain					
					Tonic					
Lamiaceae	*Nepeta leucolaena* Benth. ex Hook.f. K 129	Askuta	Herb	Leaves decoction	Stomach problem	Oral	40	0.56	53	0.74
					Constipation					
Lamiaceae	*Thymus linearis* Benth. SK 130	Tumburu	Herb	Whole plant infusion	Pneumonia	Oral	42	0.59	57	0.80
					Cough, cold					
					Respiratory disorders					
Lamiaceae	*Prunella vulgaris* L. SK 131	Harswa	Herb	Leaves decoction	Stomachache	Oral	30	0.42	24	0.33
					Sore throat					
					Diuretic					
					Tonic					
Ericaceae	*Rhododendron anthopogon* D. Don. SK 132	Chauman	Shrub	Leaves infusion	Cough, cold lung disorders	Oral	36	0.50	55	0.77
				Flower juice	Inflammations					
Onagraceae	*Epilobium latifolium* L. SK 133	Pondol	Herb	Flower paste	Itching pimples	Topical	37	0.52	28	0.39
				Leaves decoction	Skin problems	Oral				
					Antidote					
					Febrifuge					
Papilionaceae	*Astragalus psilocentros* Fisch SK 134	Sokhrus	Subshrub	Root Leaves infusion	Teeth cleaning	Oral	51	0.71	81	1.14
					Stomach problems					
Papilionaceae	*Sophora mollis* Graham SK 135	Khakhul	Shrub	Seed paste	Hepatitis	Topical	61	0.85	90	1.26
Plantaginaceae	*Plantago lanceolata* L. SK 136	Sman hrswa	Herb	Flower infusion	Diarrhea	Oral	29	0.40	48	0.67
					Dysentery					
				Leaves decoction	Asthma/breathing problem	Oral				
					Bronchitis					
					Gastritis					
Polygonaceae	*Bistorta amplexicaulis (*D.Don.) Greene SK 137	Onbu	Herb	Root infusion	Sore throat	Oral	21	0.29	24	0.33
					Laryngitis					
					Tonic					
Polygonaceae	*Oxyria digyna* (L.) Hill. SK 138	Span harswa	Herb	Leaves decoction	Jaundice	Oral	27	0.38	21	0.29
					Dysentery					
					Gastritis					
Polygonaceae	*Polygonum affine* D. Don. SK 139	Strin mindoq	Herb	Root decoction	Lung disorder	Oral	23	0.32	38	0.53
				Flower infusion	Fever, flu	Oral				
					Expel worms					
Polygonaceae	*Rheum australe* D. Don SK 140	Lachu	Herb	Leaves and stem infusion	Hepatitis B	Oral	22	0.30	34	0.47
					Appendicitis					
					Dysentery					
					Constipation					
				Root poultice	Swellings	Topical				
					Burns					
Primulaceae	*Primula denticulata* Smith SK 141	Daoo	Herb	Leaves infusion	Diabetes	Oral	28	0.39	44	0.61
					Urinary ailments					
				Root poultice	Opthalmia	Topical				
					Wound healing					
Ranunculaceae	*Aconitum violaceum* Jacquem. ex Stapf SK 142	Booma	Herb	Root decoction	Leprosy	Oral	34	0.47	48	0.67
					Asthma/breathing problem					
					Snake bite					
					Antidote febrifuge					
Ranunculaceae	*Caltha alba* Jacquem*c* SK 143	Pilling	Herb	Root paste	Toothache	Topical	32	0.45	40	0.56
					Muscular pain					
Ranunculaceae	*Delphinium brunonianum* Royle SK 144	Makhoting	Herb	Aerial parts infusion	Pneumonia	Oral	38	0.53	49	0.69
					Headache					
					Stomachache					
				Seed poultice	Healing of injuries	Topical				
Rosaceae	*Potentilla argyrophylla* Wall. ex Lehm. var. *atrosanguinea* ( G.Lodd. ) Hook.f. SK 145	Serfo harswa	Herb	Whole plant paste	Wounds	Topical	16	0.22	18	0.25
Rosaceae	*Potentilla bifurca* L. SK 146	Tarqan	Herb	Flower infusion	Diarrhea	Oral	39	0.54	26	0.36
Rosaceae	*Potentilla salesoviana* Steph. SK 152	Karfo mindoq	Herb	Flower infusion	Stomachache	Oral	10	0.14	17	0.23
					Fever					
					Cough, cold					
Rosaceae	*Rosa brunonii* Lindl. SK 147	Siya	Shrub	Bark infusion	Blood purification	Oral	63	0.88	105	1.47
Saxifragaceae	*Bergenia stracheyi* (Hook. f. & Thorns.) Engl. SK 148	Khichlay	Herb	Leaves infusion	Stomachache	Oral	44	0.61	52	0.73
					Diuretic					
				Root poultice	Tonic	Topical				
					Opthalmia					
					Wounds, cuts					
Saxifragaceae	*Bergenia ciliata* (Haw.) Sternb. SK 149	Shaphus	Herb	Leaves juice	Diarrhea	Oral	41	0.57	35	0.49
					Asthma/breathing problem					
				Seed paste	Urinary problems	Topical				
					Opthalmia					
					Boils					
Scrophulariaceae	*Pedicularis cheilanthifolia* Schrenk SK 150	Serfo spanthing	Herb	Leaves decoction	Stomachache	Oral	14	0.19	26	0.36
					Tonic					
Scrophulariaceae	*Pedicularis pectinatiformis* Bonati SK 151	Sunpo spanthing	Herb	Leaves infusion	Body ache	Oral	12	0.16	15	0.21
					Sedative					

*FC = Frequency citation, **RFC = Relative frequency of citation, ***∑Ui = sum of all uses mentioned by each informant for a species, ****UV = Use values.

### Data on quantitative ethnomedicinal uses

**Figure 5:**
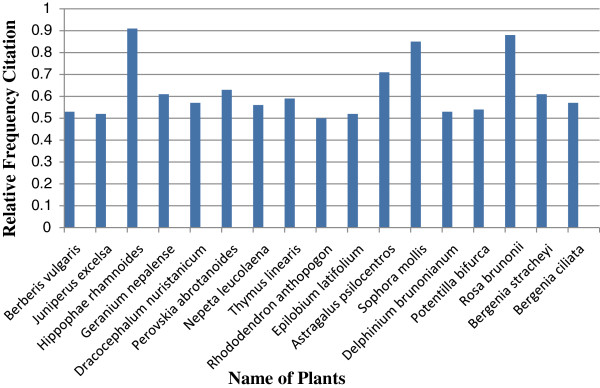
Plant species with highest Relative Frequency of Citation (RFC).

**Figure 6:**
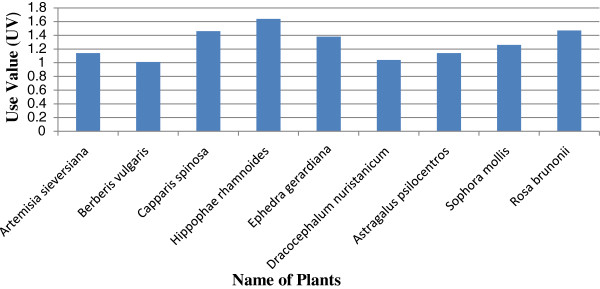
Plant species with highest Use Value (UV).

Quantitative value indices were calculated in this study to analyze the ethnomedicinal information. Figure 5 show the 17 most cited plants known by a majority of the informants for medicinal uses. The highest value of RFC ranked the *Hippophae rhamnoides* (0.91) first, followed by *Rosa brunonii* (0.88) and *Sophora mollis* (0.85) as second and third respectively. These positions correspond to the fact that the plants were reported by highest number of informants and RFC directly depends on the number of informants mentioning use of this plant (FC). Figure 6 shows the 9 most popular medicinal plants with highest use value reported by the informants. As shown in Figure 6, *Hippophae rhamnoides* has the highest use value (1.64) followed by *Rosa brunonii* (1.47) *Capparis spinosa* (1.46). The fruit of *Hippophae rhamnoides* plant is extensively used as a cure in arthritic pain, cough, relieve skin inflammation in eczema and a remedy for heart problems, ulcer, jaundice and urinary disorders. However, some additional uses of this species were also reported in previous ethnobotanical studies in other parts of Pakistan [37]. *Hippophae rhamnoides*as the characteristic and dominant species of the Skardu valley has an important role in constructing human-nature interrelationship in the area. Local people living in this valley have used different parts of this plant for various purposes of medicinal, fuel wood, household making, ornamental and decorative purposes. There is no study as such conducted by previous workers, particularly in the Skardu valley from our region, in which the RFC and UV values are calculated. The articles in which the cultural index (CI) is calculated were examined, it is seen that there is a clear difference in the type of most cited species, for example, in the study by Abbasi et al. [38]*Ficus carica* and *Ficus palmata* were the most cited species with the highest (CI) as compared to our report species *Hippophae rhamnoides* and *Rosa burnonii.* RFC and UV values obtained from the reported species indicate the degree of indigenous knowledge shared by local people regarding the use of medicinal plants in the treatment of the various ailments. These recorded values of RFC and UV were found to be higher in case of some important medicinal species, which could be attributed to the trend of utilization of herbal drugs in the valley.

The value of RFC ranges from 9 percent to 91 percent in the medicinal use of plants/herbs. The former is linked to *Jurinea Dolomiaea* from the family Asteraceae while the latter is associated with *Hippophae Rhamnoides* from family Elaeagnaceae. However, on average, the relative frequency citation is 39 percent. Likewise the UV of medicinal plants ranges from 0.12 to 1.64 which shows least relative importance of *Jurinea Dolomiaea* from the family Asteraceae to the highest importance for *Hippophae Rhamnoides* from family Elaeagnaceae. These findings are consistent with that from RFC. Keeping these configurations in view, it can be itemized that 64.39 percent inhabitants of the study area are dependent for medication upon these indigenous plants.

**Figure 7:**
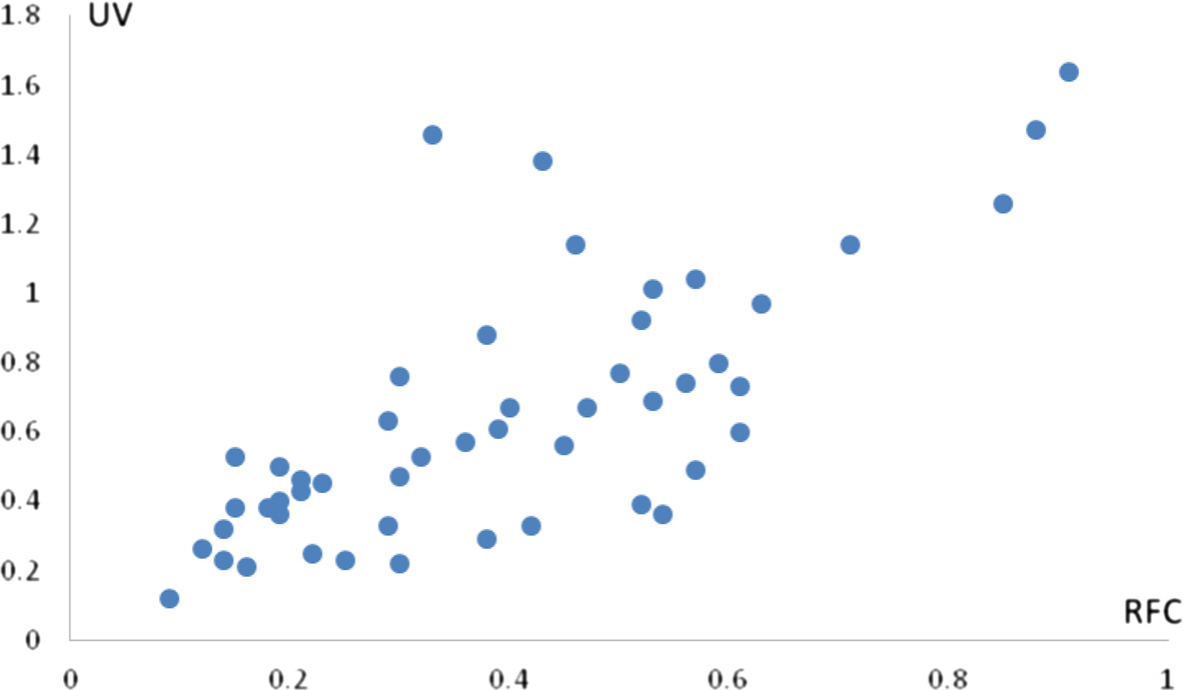
Association between Relative Frequency Citation and Use Value.

The Pearson correlation coefficient between RFC and UV was 0.732 with P-value less than 1 percent, which provide the evidence of a high positive significant association between the local importance of each species and relative importance of the use of plants. It implies that more use of species of the informants tend to increase the number of usable medicinal plants. The fact that RFC and UV are strongly positively correlated means that their patterns across species match. However, some species may have high RFC and UV while others have low values. The degree to which RFC and UV varies across species was measured numerically by r^2^ which states that around 54 percent variation in RFC can be explained by that of UV. Thus, the findings imply the empirical robustness among these two indices (Table 3). These findings are further supported by a graph drawn in the shape of the scattered plot which reflects a strong positive relationship between RFC and UV (Figure 7).

**Table 3:** Summary Stats for Relative Frequency Citation (RFC) and Use Value (UV)

	**Mean**	**Standard deviation**	**Minimum**	**Maximum**
RFC	.3944	.20452	.09	.91
UV	.6406	.37059	.12	1.64
**Association between RFC and UV by using Pearson Correlation Method**				
r	0.732 (0.000)*			
r^2^	0.536			

*Figure in parenthesis is P-values for significance of correlation coefficient.

### Comparative analysis with ethnobotanical literature

In comparative analysis of ethnomedicinal use of plants in Skardu valley and its neighboring districts, the rest of Pakistan, neighboring countries and similar studies at world level, eight published studies which meet scientific ethnobotanical standards were used. This comparison shows that there is a high similarity index between our study and ethnobotanical studies of [39,40] which is in agreement that Asteraceae (7-species), Lamiaceae (6-species), Rosaceae (4-species) and Polygonaceae (4-species) are the dominantly represented families, herbaceous life form being in highest percentage and leaves are the highly consumed part of plants. This similarity may be attributed to the similar flora, cultural uses and close proximity of the study areas but the species like *Hippophae rhamnoides, Rosa brunonii, Capparis spinosa, Ephedra gerardiana, Sophora mollis, Artemisia sieversiana, Astragalus psilocentros, Berberis vulgaris* and *Dracocephalum nuristanicum* with high UV does not agrees which may be due to specificity between regions. In contrast, the study of [41] in Serbian district Zlatibor which is not in close proximity, but confirms our findings on the high similarity index of family representation, life forms and part used.

Similar to our study [36,39,40,42] shows that the Asteraceae and Rosaceae have frequently represented families in the study areas.

The studies conducted in the surrounding districts and neighboring countries show the lowest indices of similarity [36,42-45] which probably could be attributed to cultural loss of ethnobotanical knowledge, change in floral diversity, leading to the fact that in each area people develop a use of the available species [46]. Quantitative ethnobotany calculations are performed, allowing us to discuss the results and their novelty value compared with other studies. This analysis shows a novelty of ethnomedicinal information suggesting further investigations into the ethnomedicinal wealth of the study area Table 1.

### Threats to medicinal plants and indigenous knowledge in Skardu Valley

Skardu valley is one of the naturally enriched regions of high mountains in the Karakorum- Himalayan ranges that make it unique with traditional cultural heritage but equally challenging to its community. The low standard of living and scattered population in high terrains consequently inaccessible or minimized the modern healthcare facilities for the majority of the population. This is the main reason behind the dependency of local people on medicinal plant resources to treat common day ailments. Besides the Karakoram Highway (KKH), the sole connection of Gilgit-Baltistan with the rest of Pakistan is often blocked due to the frequent land sliding round the year and intense snowfall in the winter which makes it isolated physically. It forces the local community to rely on native biodiversity to meet their daily needs. The people of the valley largely depend on wild plants for fuel, food supplements, medicine, construction material, nutrients and livestock feed. The majority of the people is engaged in agriculture, animal husbandry and forests related works. The vegetation in the valley is mostly inferior scrubs while herbs are abundant in alpine and subalpine pastures. The flora of fragile alpine meadows has been overexploited for traditional medicine because medicinal plant collectors invariably uproot the entire plant and due to this, the regrowth of some very important medicinal plants is retarded [37,47]. There is a dramatic degradation of habitat due to collection of fuel wood and shrubs to meet the domestic energy requirement as the temperature drops below −25°C in winter (November- February) and there is no alternate source. The ruthless use of valuable medicinal plants of the grazing animals is indeed a great injustice. Pastures and rangelands are used for livestock herding by the local communities on a periodic basis like high pastures are used in summer and low rangelands in autumn. During the course of study, the informants were selected randomly from the tribal communities based on their rich knowledge with long experience in utilization of medicinal plants and most of the informants belonged to or above 60 years age. In this survey, we observed that the wealth of knowledge is rapidly vanishing due to the death of elderly rural people. Due to this, transfer of indigenous knowledge from generation to generation is now endangered in this area and tends towards disappearance. Preservation of this stock of knowledge is highly important for the socioeconomic prosperity of the region. On one hand, the community can be motivated to be a party of conserving their precious resources while on the other hand experts engaged in the policy making can realize to make the flora of this region in the lime light.

## Conclusion

This study reports the first quantitative ethnomedicinal survey in the nine selected sites of Skardu valley of Northern Pakistan. Among 50 plant species belonging to 25 reported families, Asteraceae and Lamiaceae are the most used families in the area. The leaves are the favored part of local users. The most treated illnesses of the Skardu valley using medicinal plants are grouped into 33 pathological disorders. The most popular medicinal plants of the valley known by the local communities includes *Hippophae rhamnoides, Rosa brunonii, Capparis spinosa, Ephedra gerardiana, Sophora mollis, Artemisia sieversiana, Astragalus psilocentros, Berberis vulgaris* and *Dracocephalum nuristanicum* based on their highest UV and RFC values. The Pearson correlation coefficient between RFC and UV was 0.732 with P-value less than 1 percent, which provide the evidence of a high positive significant association between the local importance of each species and relative importance of the use of plants. In this way, we have compiled significant baseline data regarding indigenous knowledge about the native medicinal plants for treating common ailments is now ready to be further investigated phytochemically and pharmacologically which leads to natural drug discovery and development. Meanwhile, the medicinal plant flora of Skardu valley are threatened by major factors such as habitat degradation, grazing, expansion of new agricultural lands, and unsustainable picking of herbal plants to generate income. Practical steps should be taken immediately to ensure the inclusion of relevant flora within conservation designations for sustainable use. This would be meant to enhance the welfare level of the people living in the Skardu valley in particular and other ecosystems of similar nature in general.

## Competing interests

The authors declare that they have no competing interests.

## Authors’ contributions

AB: This work is part of my PhD thesis; I carried out ethnomedicinal survey in highly remote areas of Pakistan-Skardu valley. MA and MZ supervised this ethnobotanical project and helped in plant identification. MA and SS helped in editing the manuscript and quantitative data analysis. MAA and TBH provided technical expertise in compiling data into the draft. AS, MAA and MA helps in statistics. All the authors read and approved the final manuscript.
